# SiPM-based optical sensing system for GIS partial discharge detection

**DOI:** 10.1371/journal.pone.0349200

**Published:** 2026-05-14

**Authors:** Shiman Lin, Yusheng Chen, Deyu Qu, Yijia Ren, Xin Li, Jiangnan Liu

**Affiliations:** Guangzhou Power Supply Bureau, Guangdong Power Grid Co., Ltd., Guangzhou, China; Donghua University, CHINA

## Abstract

The silicon photomultiplier (SiPM)-based optical detection method, which provides strong resistance to electromagnetic interference and high sensitivity, is attracting increasing attention. However, most studies have been conducted on laboratory simulation platforms, and few studies have systematically investigated SiPM-based partial discharge (PD) detection in actual gas insulated switchgear (GIS) equipment. In addition, the photon detection efficiency (PDE) of SiPMs varies with photon wavelength, and current studies lack a theoretical basis for the selection of SiPMs. To address this issue, an experimental platform for GIS gas discharge analysis was established in this study to systematically investigate the emission spectral characteristics of SF_6_ gas discharge at different discharge intensities, pressures, and temperatures. On this basis, a SiPM-based optical sensing system adapted for GIS applications was developed. Ultimately, a PD detection experiment was conducted on an actual 110 kV GIS platform. The experimental results indicate that the detection signal-to-noise ratio of the developed SiPM-based optical detection system is approximately 5.8 dB higher than that of the high-frequency current transformer and that the minimum detectable discharge quantity is below 18 pC. These results highlight the potential of SiPM-based optical detection technology for GIS PD detection.

## I. Introduction

Gas insulated switchgear (GIS) is a critical component in power systems; its reliability is essential to the stable operation of the system [1]. However, long-term operational experience indicates that various defects (e.g., free metal particles and sharp protrusions) introduced during GIS manufacturing, installation, and operation often cause partial discharges (PDs), which may further develop into flashover or breakdown [2,3], resulting in occasional GIS insulation faults. Therefore, improving PD detection is critical for enabling early fault warnings.

Partial discharge (PD) in power equipment produces various physical phenomena, including electromagnetic, acoustic, and optical signals, enabling detection via pulse current, ultrahigh frequency (UHF), ultrasonic, and optical methods [4–7]. The pulse current and UHF methods offer high sensitivity but are susceptible to electromagnetic interference, leading to false positives [8]. Although ultrasonic detection is immune to electromagnetic interference, the sensitivity of acoustic sensors is heavily limited by severe acoustic attenuation [9]. Additionally, electromagnetic sensors are easily affected by the complex electromagnetic interference within GIS, limiting their application in PD detection [10,11]. In contrast, optical detection, which relies on discharge photon emission, provides strong immunity to electromagnetic interference and high sensitivity, making it a promising approach for GIS PD monitoring.

After decades of development, the optical signal detection of GIS PD currently utilizes three typical types of sensors: vacuum photomultiplier tubes (PMTs), fluorescent fiber sensors, and silicon photomultiplier (SiPM) sensors [12]. For example, Li et al. [13] experimentally investigated the performance of PMTs in detecting the discharge of suspended metallic particles on the surface of GIS basin insulators; their results indicated a correlation between PMT and UHF detection signals. Chen et al. [14] confirmed the applicability of phase-resolved PD patterns based on PMTs in the evaluation of discharge severity. However, the large physical size of PMTs and their reliance on a high-voltage power supply of several hundred volts render installation and operation difficult. Consequently, these studies can only be conducted under laboratory conditions and cannot be readily extended to GIS equipment in the field. To address this issue, researchers have proposed discharge photon detection methods based on fluorescent optical fibers [15–17], which utilize discharge photons to excite the energy-level transitions of rare-earth ions to generate fluorescence for discharge sensing. However, this method is significantly affected by transmission losses and ambient temperature variations, leading to practical engineering challenges such as low detection sensitivity and poor stability.

Owing to the rapid development of optoelectronic theory and devices, SiPM-based optical discharge detection technology is attracting increasing attention from researchers. The SiPM is a microcell array that integrates thousands of single-photon avalanche diodes (SPADs) and series resistors on a chip area at the mm^2^ scale and exhibits single-photon-level detection sensitivity. In addition, it operates at relatively low bias voltage, making it well suited for GIS partial discharge detection. A performance comparison of the aforementioned optical detection sensors is presented in Table 1.

**Table 1:** Performance Comparison of Three Types of Optical Detection Sensors.

Index	PMT	Fluorescent fiber	SiPM
Gain	≥10^6^	1	≥10^6^
Operating voltage	~1000 V	0	30 V
Cost	about 3000 $	about 100 $	about 1000 $
Size	>10cm	～mm	～mm
Temperature sensitivity	Low	High	Low

In 2017, Ren et al. [18] conducted a comparative study on the performances of PMTs and SiPMs in detecting three typical defects, i.e., protrusion, floating, and particle defects; and their results indicated that SiPMs and PMTs have comparable detection sensitivities. In another study [19], the detection performance of SiPMs and the pulse current method were compared, and the results indicated that SiPMs are comparable to the latter in terms of the signal-to-noise ratio (SNR), pulse resolution, and linearity. Although the above studies have reported the potential of SiPMs for PD detection, they have generally been conducted on laboratory simulation platforms, and few studies have systematically investigated SiPM-based PD detection in actual GIS equipment. In practical GIS structures, photon propagation can be significantly affected by shielding from internal components such as conductors and grading rings, which may limit the effective collection of discharge light. Therefore, experimental validation on real GIS equipment is essential to accurately evaluate the detection capability of SiPMs for partial discharge defects. In addition, the photon detection efficiency (PDE) of SiPMs varies significantly with photon wavelength, and the sensitive wavelength ranges vary significantly among different SiPM devices. However, existing studies typically select SiPMs in an empirical manner, without establishing a quantitative relationship between the discharge emission spectrum and the detector response. This lack of a spectral–device matching framework may lead to suboptimal photon utilization and limits the detection sensitivity. Therefore, developing a physically grounded basis for SiPM selection is essential for improving the performance of optical PD detection systems.

## Ⅱ. Materials and methods

To address this research gap, an experimental platform for GIS gas discharge analysis was established in this study, and the spectral characteristics of discharge photons under various GIS operating conditions were experimentally determined. A SiPM sensor system suitable for GIS PD detection was developed, and a packaging scheme was designed in accordance with the structural characteristics of GIS. In addition, an experimental platform was constructed on the basis of an actual 110 kV GIS bus to verify the ability of the developed system to detect PD defects in actual GIS equipment.

## III. Results

### A. GIS PD emission spectral characteristics

The typical characteristics of the discharge emission spectrum of a GIS under rated operating conditions were first analyzed in this study. In addition, the internal temperature field of a GIS varies due to fluctuations in the load current during its operation, and the SF_6_ filling pressure also differs among individual chambers. The SF_6_ emission spectra were analyzed at different temperatures and pressures to elucidate the influence of various operating conditions on the GIS PD emission spectra.

### B. Experimental platform

**Fig 1:**
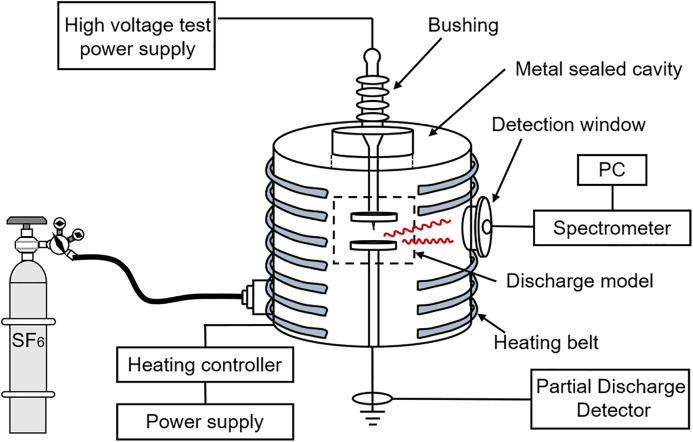
Schematic of the Experimental Platform for GIS Gas Discharge Analysis.

The experimental platform for GIS gas discharge analysis is shown in Fig 1. The platform consists of a high-voltage test power supply, a metal-sealed chamber, a discharge model spectral acquisition system, and a discharge detection system. The spectral acquisition system guides the optical signal out of the metal-sealed chamber through an ultraviolet (UV)-resistant optical fiber and transmits it to a spectrometer, which has a detection range of 200–980 nm and a wavelength resolution of 2 nm. The discharge defect has a needle-plate structure with an interelectrode gap of 10 mm and a needle electrode tip curvature radius of ~0.3 mm. The test power supply applies high voltage to the needle electrode through the bushing, and the plate electrode is grounded. The discharge detection system includes a high-frequency current sensor and a PD detector, enabling quantitative evaluation of PD intensity. In addition, heating bands are installed outside the metal chamber, which heat the chamber to simulate the temperature field in actual GIS equipment.

### C. Discharge emission spectra under rated operating conditions

**Fig 2:**
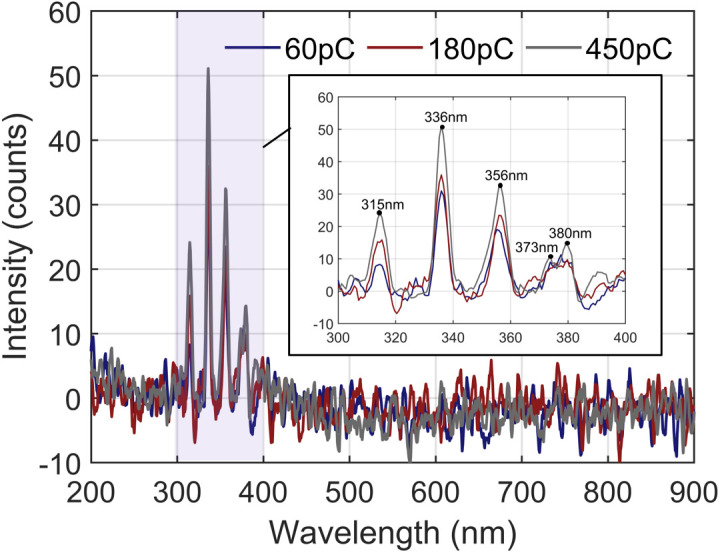
SF_6_ Discharge Emission Spectra at Different Discharge Levels.

Based on the experimental platform shown in Fig 1, discharge emission spectrum tests were conducted under rated operating conditions. Before the experiment, the chamber was evacuated and filled with high-purity SF_6_ to 0.4 MPa. At room temperature (~25 °C), an alternating current (AC) high voltage was applied to the needle-plate defect model to generate a stable discharge. The discharge photon spectra were recorded at average apparent discharge capacities of 60 pC (weak discharge), 180 pC (moderate discharge), and 450 pC (violent discharge). The discharge emission spectra at different discharge levels are shown in Fig 2.

As shown in Fig 2, when discharge occurred in the GIS gas medium, the resulting photon spectrum had five characteristic peaks at 315 nm, 337 nm, 356 nm, 373 nm, and 380 nm, all concentrated in the near-ultraviolet (UV) region. In addition, as the discharge intensity increased, the spectral peak positions remained unchanged, whereas the spectral intensity increased. These results indicate that regardless of the discharge intensity, the mechanism of photon emission in the GIS gas medium remains unchanged. The theoretical explanation of this mechanism is as follows.

Under the strong electric field at the needle tip, the free electrons in the gas medium accelerate and collide with the gas molecules, causing the particles to undergo stimulated emission and release energy in the form of photons with the emission wavelength *λ*, which is expressed as [20]:


$$\begin{array}{c} \lambda =\frac{hc}{E_{q}-E_{p}} \end{array}$$ (1)

where *h* represents Planck’s constant; *E*_*q*_ and *E*_*p*_ denote the binding energies of the electron orbitals corresponding to the low- and high-energy levels, respectively; and *c* indicates the speed of light in vacuum.

As expressed in this equation, the discharge emission spectrum is determined by the transitions between electronic states in gas molecules, which are accompanied by changes in vibrational energy levels. In accordance with the Spectral Database for Organic Compounds (SDOC), the characteristic peaks of the stimulated emission spectrum of the second positive band of the N_2_ molecule (Table 2) are consistent with the experimental results. Therefore, during gas discharge in GIS, N_2_ is the dominant molecule that produces stimulated emission.

**Table 2:** Comparison of the Spectral Test Results and the SDOC Database Results.

Measured results (nm)	SDOC data (nm)
315	315.87
336	337.13
356	357.69
373	371.05
380	380.49

The current standards require that the vacuum level of the GIS before filling with SF_6_ is < 133 Pa, which cannot achieve absolute vacuum. In addition, the SF_6_ used also contains trace amounts of N_2_ as an impurity. Therefore, N_2_ is inevitably retained inside the GIS. Although SF_6_ has more molecules than N_2_ does, its vibrational–translational relaxation effect is significant, and most of the electron collision energy is converted to thermal energy rather than emitted as photons. Therefore, the spectral characteristics of the GIS discharge emission are determined by the small amount of residual N_2_.

### D. Effect of pressure on the discharge emission spectrum

**Fig 3:**
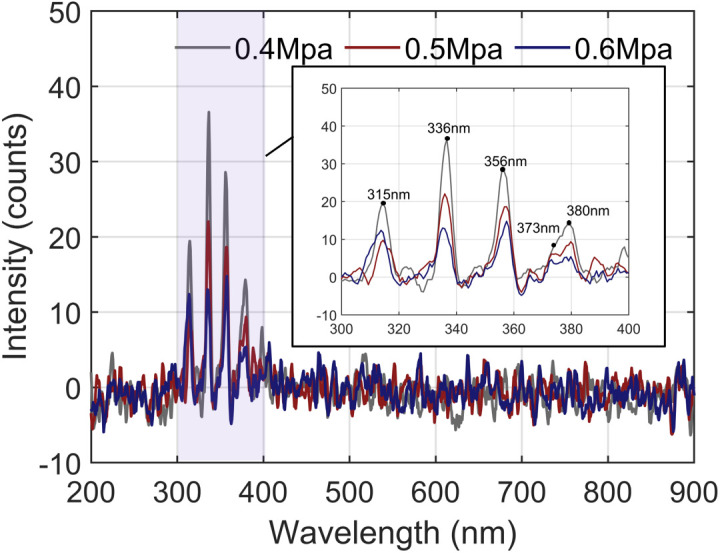
SF_6_ Discharge Emission Spectra at Different Gas Pressures.

Based on the above findings, the number of SF_6_ molecules may affect the stimulated emission behaviour of N_2_. Therefore, further investigation of the GIS discharge emission spectra at different SF_6_ filling pressures is needed. In accordance with the actual operating conditions of the GIS equipment, an AC high voltage of 40 kV was applied to the discharge defects at chamber pressures ranging from 0.4 MPa to 0.6 MPa to produce stable discharges. The spectra obtained at different pressures are shown in Fig 3.

As shown in Fig 3, at different pressures, the peak positions of the SF_6_ discharge emission spectra remained unchanged; that is, they remained concentrated in the near-UV region. These results indicate that the number of SF_6_ molecules does not affect N_2_ as the dominant stimulated emission particle. However, as the gas pressure increases, the collision of electrons under the same electric field is suppressed, reducing both the stimulated emission intensity of N_2_ and the spectral amplitude.

### E. Effect of temperature on the SF_6_ discharge emission spectra

**Fig 4:**
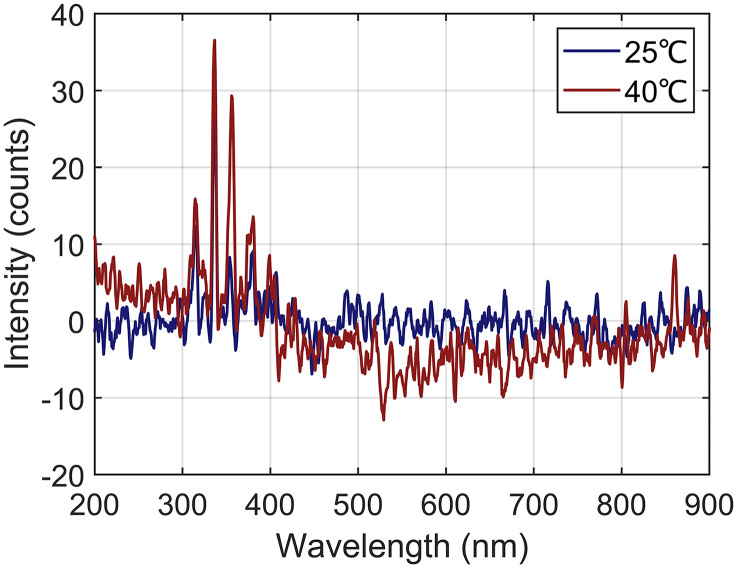
SF_6_ Discharge Emission Spectra at Different Temperatures.

Temperature affects the energy of gas molecules and thereby the behavior of stimulated emission. To quantify the variations in the emission spectra of the gas discharge within the normal operating temperature range of the GIS, the average gas temperature in the chamber was controlled to 25 °C and 40 °C by external heating, and an AC voltage of 40 kV was applied to the discharge defects. The resulting discharge emission spectra are shown in Fig 4.

As shown in Fig 4, the SF_6_ discharge emission spectra at different temperatures are generally consistent, with characteristic emission peaks still concentrated in the near-UV region. However, as the temperature increased, the spectral intensity in the near-UV region increased slightly. This finding may be attributed to the desorption of nitrogen molecules originally adsorbed on solid materials (such as insulators and metal conductors) induced by thermal effects, which increases the concentration of N_2_ impurities in the GIS. In addition, the experiment demonstrated that at 40 °C, a characteristic peak at 860 nm appeared in the near-infrared region of the gas discharge emission spectrum. This finding is associated with the excitation-induced generation of free fluorine atoms from the decomposition products of SF_6_ [21]. Overall, the gas discharge emission spectrum of GIS within the normal operating temperature range remained dominated by the stimulated emission from N_2_.

## IV. Discussion

### A. SiPM-based discharge detection system

**Fig 5:**
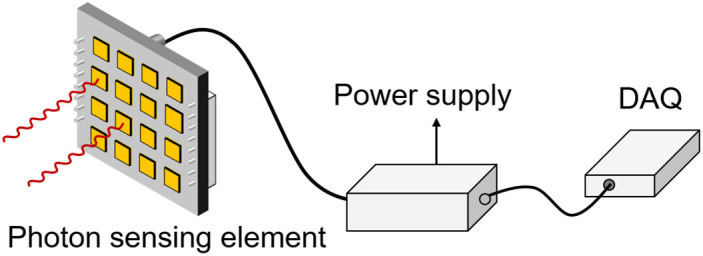
Schematic of the SiPM-Based Discharge Detection System.

Based on the GIS PD emission spectral characteristics, a SiPM-based discharge detection system can be designed. It consists of a photon sensing element, a signal conditioning circuit, and a data acquisition module (Fig 5). The photon sensing element converts the optical signal generated by the discharge to a current signal output, and the signal conditioning module converts the current signal to a voltage signal, which is recorded and stored by the data acquisition module.

**Fig 6:**
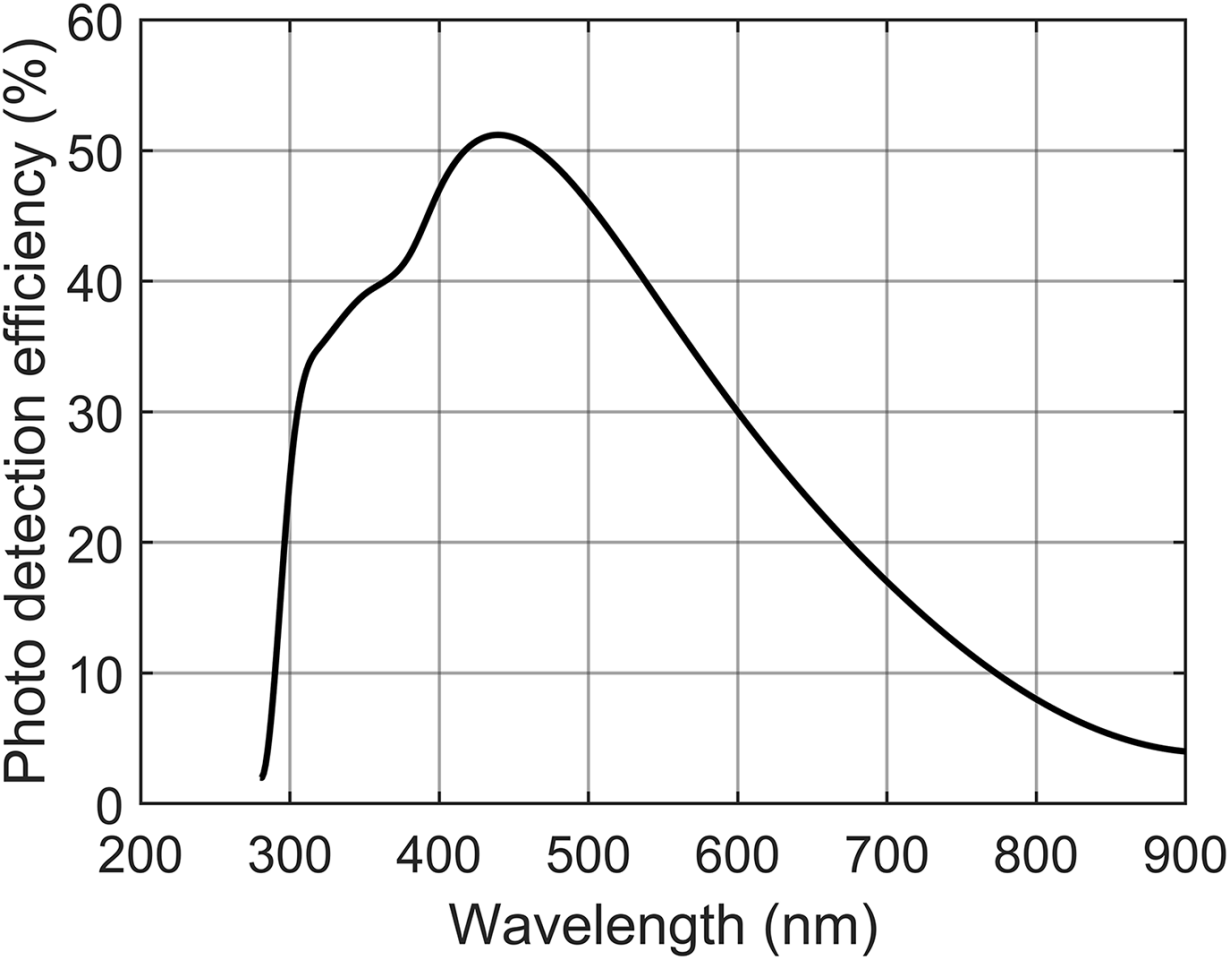
PDE Curve of the S14160 SiPM.

The preceding spectral analysis reveals that, under various typical GIS operating conditions, the wavelengths of the photons generated during SF_6_ PD are mainly in the range of 300 nm to 400 nm. Based on this physical mechanism, to achieve high-sensitivity detection of weak PD optical signals, the Hamamatsu S14160 SiPM was optimally selected as the photon sensing element in this study. As indicated by the PDE curve of the S14160 SiPM shown in Fig 6, the average PDE of this device reaches 37.8% within the characteristic wavelength range of 300 nm to 400 nm, demonstrating that its spectral response range highly matches the SF_6_ discharge emission spectrum. This not only physically ensures the efficient capture of discharge photons by the detection system but also effectively suppresses background optical noise interference from non-characteristic bands. Furthermore, the device features a photosensitive array area of 6 mm × 6 mm composed of 159565 parallel-connected microcells (with a microcell pitch of 15 μm). Such a massive number of microcells effectively circumvents the photon miscounting effect induced by dead-time, thereby endowing the sensor with an excellent capability for capturing transient multiphoton pulses [18].

**Fig 7:**
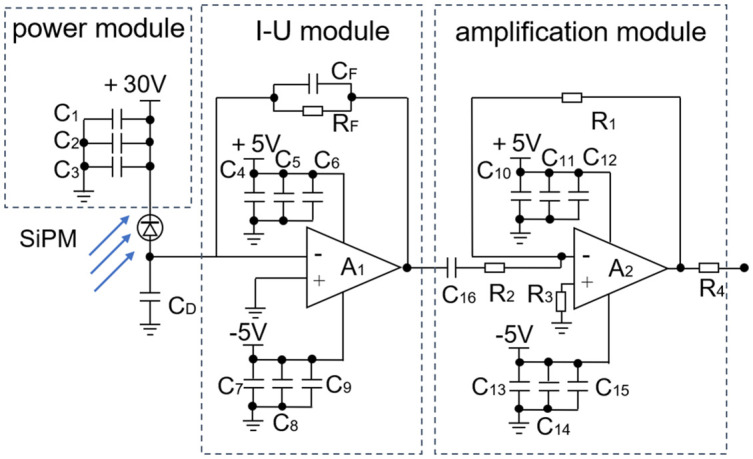
Schematic of the Signal Conditioning Circuit.

**Fig 8:**
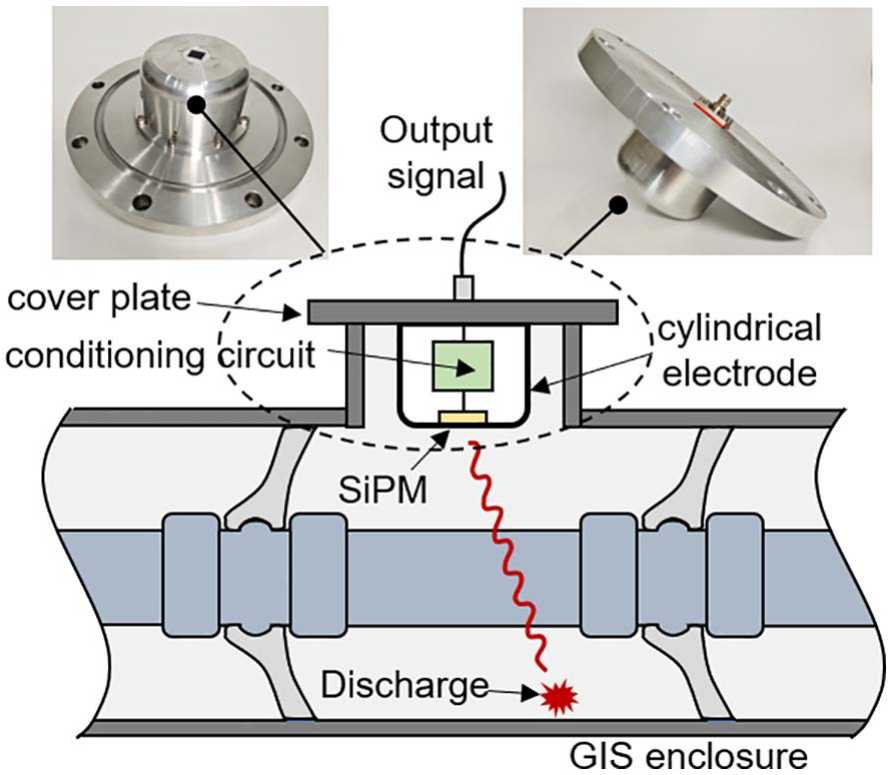
Packaging Structure of the SiPM-Based Discharge Detection System.

The output of a SiPM is a weak current signal. Therefore, the signal conditioning circuit shown in Fig 8 was designed in this study to facilitate detection. The circuit can be divided into three parts: a power supply module, a current conversion module, and a signal amplification module. The current conversion module converts the transient current pulse output by the SiPM to a voltage signal through the transimpedance amplifier A_1._ The signal amplification module amplifies the voltage signal and outputs it after impedance matching with the coaxial cable through R_4_ = 50 Ω. In Fig 7, R_F_ represents the transimpedance feedback resistor, which is used to adjust the I–U conversion gain; C_F_ denotes the feedback compensation capacitor, which is used to suppress operational amplifier resonance and prevent chip overheating; R_1_ and R_2_ are used to set the gain of the inverting amplifier; and capacitors C_1_ to C_15_ are used for decoupling and filtering to reduce the effect of the power supply ripple and noise on front-end amplification.

To enable the integrated installation of the SiPM detection system and GIS equipment, the packaging structure shown in Fig 8 was designed in this study. In this structure, the photon sensing device is embedded on the surface of a columnar electrode with a diameter of 120 mm and a height of 60 mm, and the photosensitive surface faces outward and is flush with the outer surface of the electrode. The signal conditioning circuit is placed inside the columnar electrode. The electrode and the cover plate are bolted together and grounded to ensure that the signal conditioning circuit inside the electrode is not affected by the strong electric field inside the GIS. The cover plate can be installed on the observation window of the GIS so that the SiPM can receive photon signals generated by the internal discharge of the GIS. A hermetic connector is installed on the cover plate to lead the output signal of the signal conditioning circuit to the external data acquisition module. Because the end face of the columnar electrode is flush with the inner surface of the GIS enclosure, this packaging structure does not distort the electric field distribution inside the GIS and poses no insulation risk.

It is worth mentioning that the SiPM-based sensing system operates within GIS equipment, which provides a sealed and well-controlled environment. This inherently limits external disturbances and the effect of temperature on SiPM gain and dark noise. Therefore, the proposed SiPM-based system demonstrates strong potential for reliable long-term PD monitoring.

### B. Optical pone.0349200

**Fig 9:**
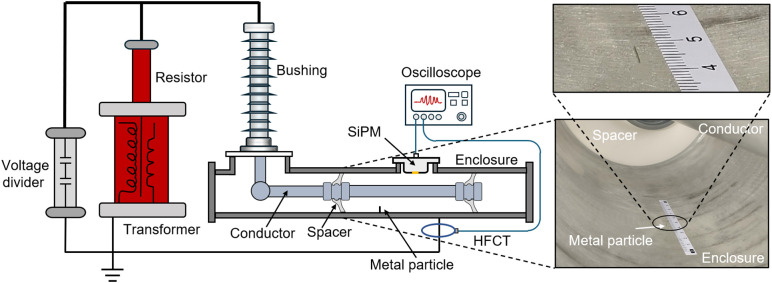
Experimental Platform for PD Detection in a 110 kV GIS.


To investigate the performance of the SiPM detection system for detecting PD defects under actual GIS conditions, an experimental platform was constructed using an actual 110 kV GIS, as shown in Fig 9. In the experimental platform, the SiPM packaging structure was mounted at the operating hand hole of the GIS enclosure via a flange, and a high-frequency current transformer (HFCT) was used to couple the pulse current generated by the PD to quantify the discharge level. A 5-mm aluminum wire with a diameter of ~0.15 mm was placed on the inner surface of the GIS enclosure to simulate a tip defect. Before the experiment, the GIS chamber was evacuated and filled with SF_6_ to 0.4 MPa. In addition, the response performance of the HFCT was calibrated to obtain the relationship between the HFCT output signal amplitude *U*_q_ (mV) and the apparent discharge quantity *Q* (pC) of the PD, as shown in the following equation:

$$\begin{array}{c} Q=1.9632U_{q} \end{array}$$ (2)

**Fig 10:**
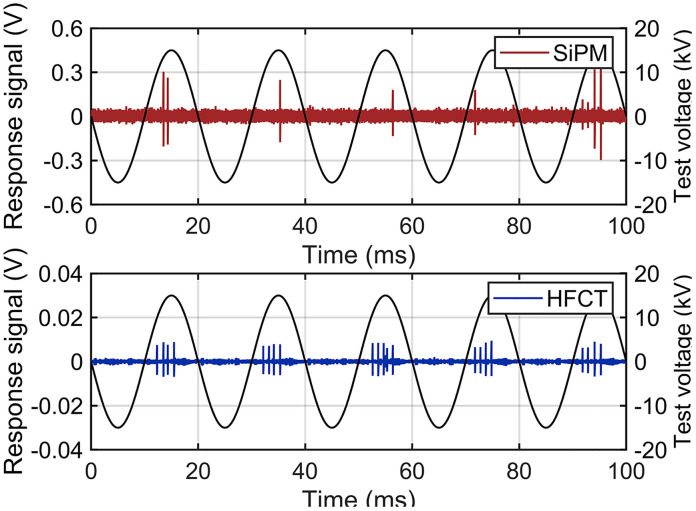
Discharge Signals Detected by the SiPM and HFCT (100 ms).

During the experiment, voltage was applied to the central conductor of GIS through a power-frequency test transformer using the step-up method (in 1 kV increments). When the peak voltage was increased to 15 kV, the SiPM sensing system and the HFCT detected the discharge signals simultaneously, and the detection results within 100 ms are shown in Fig 10.

As shown in Fig 10, the output signals of the SiPM and HFCT exhibit high temporal correlation, and the pulse signals generated by the PD can be detected in the positive half-cycle of each power-frequency cycle. However, the pulse repetition rate detected by the SiPM is lower than that of the HFCT, which may be attributed to partial shielding of discharge photons by the conductor.

**Fig 11:**
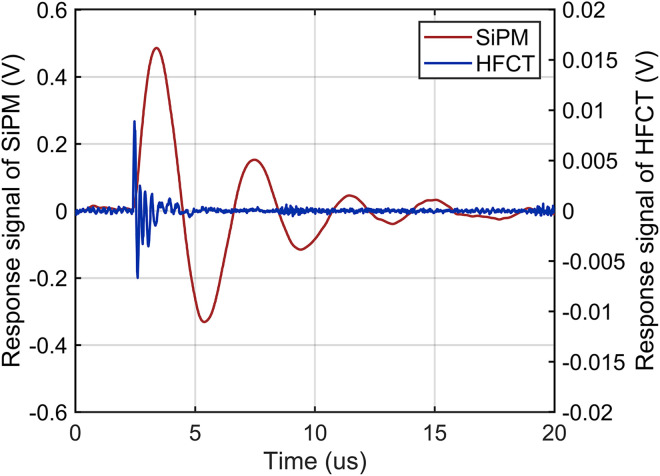
Discharge Signals Detected by the SiPM and HFCT (20 us).

**Fig 12:**
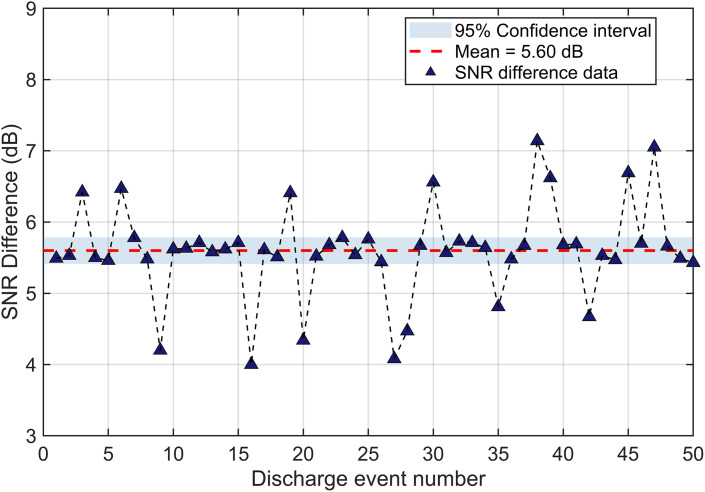
Comparison of the SNRs between the SiPM and HFCT for 50 Discharge Events.

Representative discharge response signals of the SiPM and HFCT are shown in Fig 11. Taking a typical discharge event as an example, the response signal amplitudes of the SiPM and HFCT are 501 mV and 9.1 mV, respectively. Because the background noise levels of the SiPM and HFCT are approximately 45 mV and 1.6 mV, respectively, the SNR of the SiPM discharge detection system is 20.93 dB, which is 5.8 dB higher than that of the HFCT. Furthermore, the SNRs of the SiPM and HFCT were compared over 50 discharge events, as shown in Fig 12. The results indicate that the average SNR of the SiPM-based detection system is approximately 5.6 dB higher than that of the HFCT.

**Fig 13:**
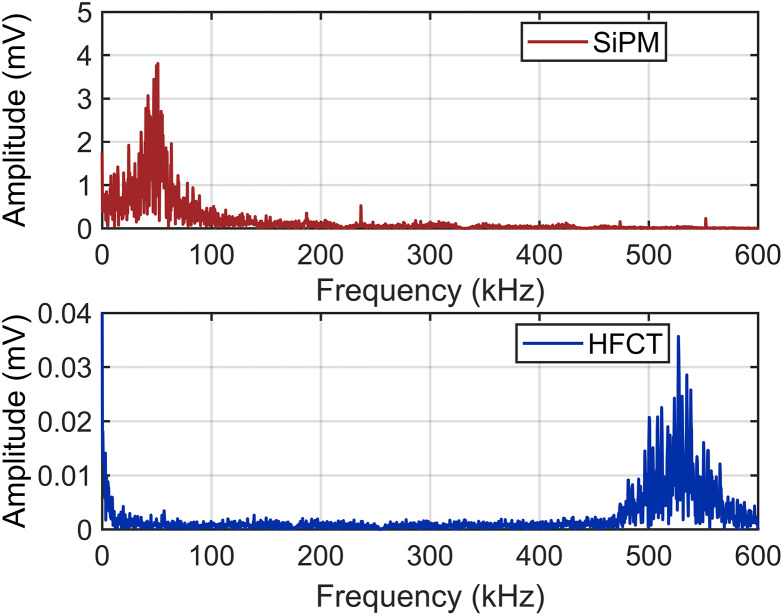
Frequency-Domain Results of the Discharge Signals Detected by the SiPM and HFCT.

In addition, as shown in Fig 11, the pulse signals detected by both types of sensors exhibit damped sinusoidal waveforms, which, however, have significantly different characteristics. The wavefront time of the HFCT response signal is 102 ns, whereas that of the SiPM is 1050 ns, a difference associated with the operating mechanisms of the sensing devices. Fourier transform was performed on the response signals of the SiPM and HFCT, and the resulting frequency-domain characteristics of the signals are presented in Fig 13.

As shown in Fig 13, the GIS discharge photon signals obtained by the SiPM-based discharge detection system were mainly below 100 kHz, whereas the pulse current signals were mainly distributed in the frequency range of 480 kHz to 600 kHz. Evidently, using SiPM for GIS PD detection facilitates both data acquisition and processing.

**Fig 14:**
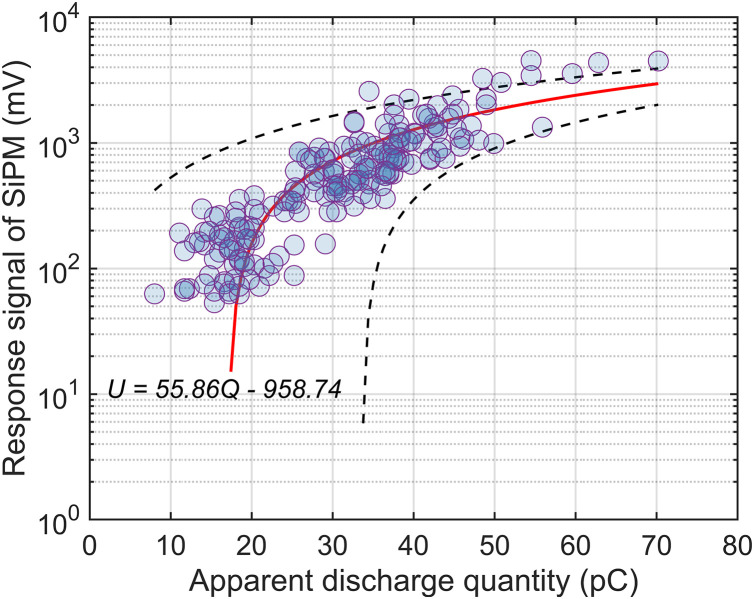
Relationship between the SiPM Response Signal and the Apparent Discharge Quantity.

In addition, the apparent discharge quantity for 200 experimental data points was calculated on the basis of the HFCT output signal and Equation (2), and hence a quantitative relationship between the SiPM response signal and PD intensity was obtained, as shown in Fig 14.

As shown in Fig 14, as the apparent discharge quantity increases, the response signal of the SiPM tends to increase overall. To quantify this trend, a linear fitting alongside a 95% confidence band was introduced. Despite the inherent dispersion of discharge events, the fitting yields an R^2^ of 0.6463, demonstrating a clear positive correlation.. This finding indicates that the SiPM-based detection results can be used to evaluate discharge severity in GIS. In addition, the background noise of the SiPM discharge detection system is approximately 45 mV, according to the scatter distribution and the fitted trend in Fig 14, the minimum detectable discharge quantity of the system is less than 18 pC.

## V. Conclusions

The SiPM-based GIS PD detection method was investigated in this study, and experimental tests were conducted. The following conclusions are reached:

1) The optical emission spectrum of GIS partial discharge is characterized by several dominant peaks in the near-UV region (300–400 nm), associated with gas discharge processes involving residual components such as N_2_. The peak positions remain stable under typical variations in discharge intensity, pressure, and temperature.
2) A spectral-characteristics-guided selection strategy for SiPM devices is established, demonstrating that sensors with high photon detection efficiency in the near-UV region enable high-sensitivity detection of weak PD optical signals.
3) Experimental validation on a 110 kV GIS platform demonstrates that an average SNR of the SiPM-based detection system is approximately 5.6 dB higher than that of the HFCT and a minimum detectable discharge below 18pC, confirming its superior sensitivity for micro-discharge detection. In addition, the detected photon signals are mainly distributed below 100 kHz, which facilitates signal acquisition and processing compared with conventional electromagnetic methods.

## Supporting information

S1 Data Raw numerical data underlying Figs 2, 3, 4, 6, 10, 11, 12, 13 and 14. (ZIP)
